# Patients and electroconvulsive therapy: Knowledge or attitudes?

**DOI:** 10.4103/0019-5545.33268

**Published:** 2007

**Authors:** Chittaranjan Andrade

**Affiliations:** National Institute of Mental Health and Neurosciences, Bangalore - 560 029, India. E-mail: andrade@nimhans.kar.nic.in

Sir,

Chavan *et al*.[1] sought to assess knowledge about and attitudes towards electroconvulsive therapy (ECT) in stable psychiatric patients and their psychiatrically healthy relatives. I am concerned that Chavan *et al*.[1] suggested correct answers for all the items in their questionnaire. This implies that all items tapped knowledge but none addressed attitudes. That this is an important issue can be understood from item 3, which states that the use of ECT leads to permanent memory loss and item 12, which states that ECT is painful. To be scored as correct, respondents were expected to disagree with both statements.

Is this scoring valid? I think not. No study has systematically examined long-term memory loss after ECT for us to assume that such memory loss does not exist. In fact, the available data suggests that at least some patients may experience extensive, protracted and possibly permanent memory disturbance after ECT. For example, Lisanby *et al*.[2] described loss of impersonal memories that was evident even two months after ECT. Weiner *et al*.[3] observed that autobiographical memories were impaired even six months after bilateral sinewave ECT. Donahue[4] described profound autobiographical memory loss which was still prominent three years after ECT. Fink[5] suggested that about one in 200 patients treated with ECT can expect to suffer such severe memory disturbance.

And does ECT cause pain? It certainly might. Patients may reasonably be expected to consider the injection of the ECT premedication noxious and many patients do experience headache or bodyache for several hours after an ECT treatment.

Items 3 and 12 therefore elicit attitudes more than knowledge; this is even more true for item 2, which states that ECT is an inhuman treatment. To some, it is; to others, it is not. There cannot be a correct position for an attitude; for example, there are proponents for and against the death sentence and who has the right to say which stance is correct? It is therefore necessary to separate items reflecting knowledge from those reflecting attitudes and, to view attitudes as positive and negative (rather than right or wrong), much as we did in a study which has now become a landmark reference in the field.[6] Other methodological issues also merit consideration and we have dealt with these elsewhere.[7]
